# Neonatal renal and inferior vena cava thrombosis associated with fetal thrombotic vasculopathy: a case report

**DOI:** 10.1186/s13256-017-1414-0

**Published:** 2017-08-28

**Authors:** Lorenzo Giacchetti, Martina De Gaudenzi, Andrea Leoncini, Elisabetta Ferrucci, Valdo Pezzoli, Manuela Albisetti

**Affiliations:** 1 Department of Pediatrics, Regional Hospital of Lugano, Via Tesserete 46, 6900 Lugano, Switzerland; 2 Department of Pediatrics, University of Pavia, Pavia, Italy; 3 Department of Radiology, Regional Hospital of Lugano, Lugano, Switzerland; 4 Division of Hematology, University Children’s Hospital, Zurich, Switzerland

**Keywords:** Neonate, Venous thrombosis, Fetal thrombotic vasculopathy, Umbilical cord

## Abstract

**Background:**

Fetal thrombotic vasculopathy is a described placental diagnosis associated with adverse perinatal outcomes. It may also predispose children to somatic thromboembolic events. As far as we know, this is the first case of inferior vena cava thrombosis associated with fetal thrombotic vasculopathy in a completely asymptomatic newborn.

**Case presentation:**

We report the case of an asymptomatic, full-term Turkish male neonate delivered at 39 weeks of gestation diagnosed as having thrombosis of the renal vein and inferior vena cava. Diagnosis was guided only by the presence of edematous umbilical cord with macroscopic signs of clotting and, subsequently, microscopic features of the placenta, suggesting fetal thrombotic vasculopathy.

**Conclusions:**

Thrombosis of the renal and inferior vena cava in our healthy, asymptomatic full-term neonate is clearly associated with fetal thrombotic vasculopathy. The diagnosis of thrombosis in this neonate was incidental. This suggests that fetal thrombotic vasculopathy may cause unrecognized neonatal thrombosis. Untreated neonatal thrombosis may later compromise growth and function of the involved organs; therefore, maintaining a high index of suspicion based on thrombotic vasculopathy is paramount.

## Background

The vast majority of neonatal thromboembolic events (TEs) are related to catheterization and most of their discoveries are secondary to clinical manifestations [1]. Fetal thrombotic vasculopathy (FTV) is a recently described placental diagnosis associated with adverse perinatal outcomes. Neonates from mothers who have FTV are more likely to have intracranial hemorrhage, coagulopathy, neurological impairment, growth retardation, and evidence of systemic thrombosis/vasculopathy [2, 3].

We report the case of an otherwise healthy asymptomatic neonate who was discovered to have thrombosis of the inferior vena cava (IVC) on imaging. An ultrasound was performed on the baby due to edema and macroscopic signs of thrombosis observed in the umbilical cord. The placenta histologically showed signs of FTV.

## Case presentation

A full-term male Turkish neonate was delivered at 39 weeks of gestation by an urgent caesarian section due to fetal heart rate decelerations, after a failed attempt of vacuum-assisted vaginal delivery. The pregnancy of his 30-year-old gravida 4 para 2 mother had been uneventful. She had previously experienced two spontaneous abortions of unclear etiology. Their family history was negative for coagulopathies.

The neonate was asymptomatic at birth, showing a good transition to extrauterine life. An initial physical examination revealed no pathologic findings; Apgar scores were 7, 9, and 9 at 1, 5, and 10 minutes of life, respectively, and arterial cord pH was 7.24. His body weight was 3420 grams, body length 52 cm, and head circumference 34 cm. A neurological examination revealed no abnormalities.

**Fig. 1:**
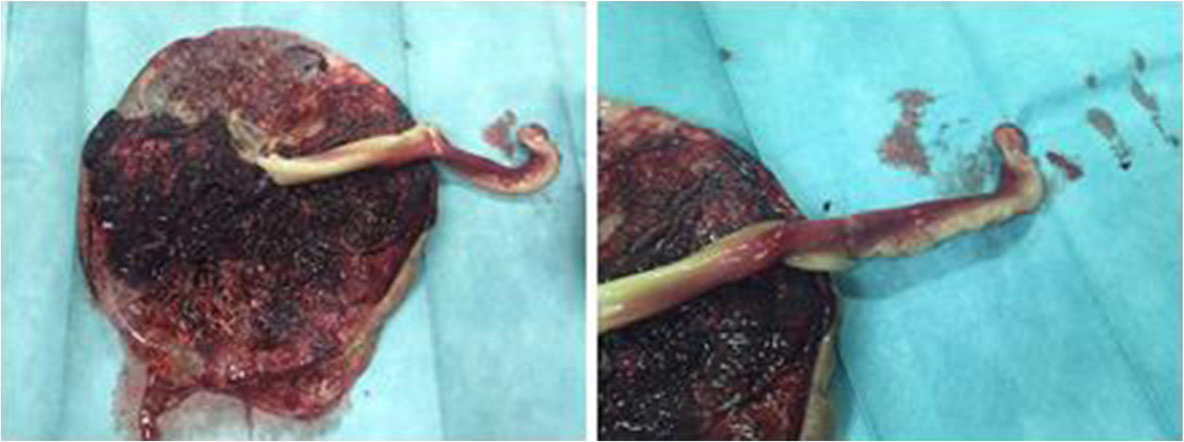
Placenta and umbilical cord at birth

**Fig. 2:**
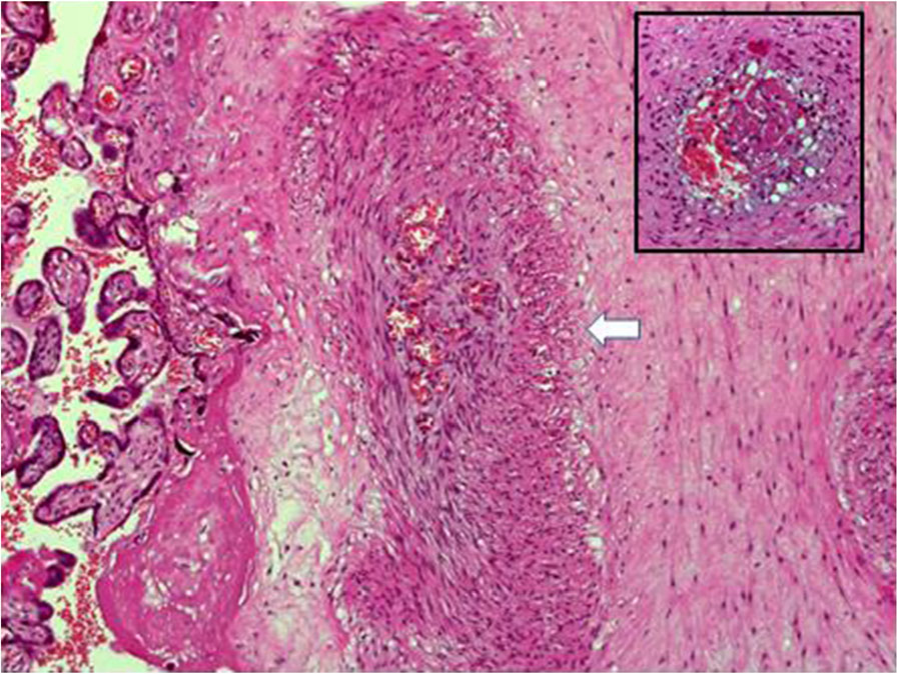
Organized thrombus and recanalized stem villus (*large photo arrow*); thrombus not completely organized (*top right*)

**Fig. 3:**
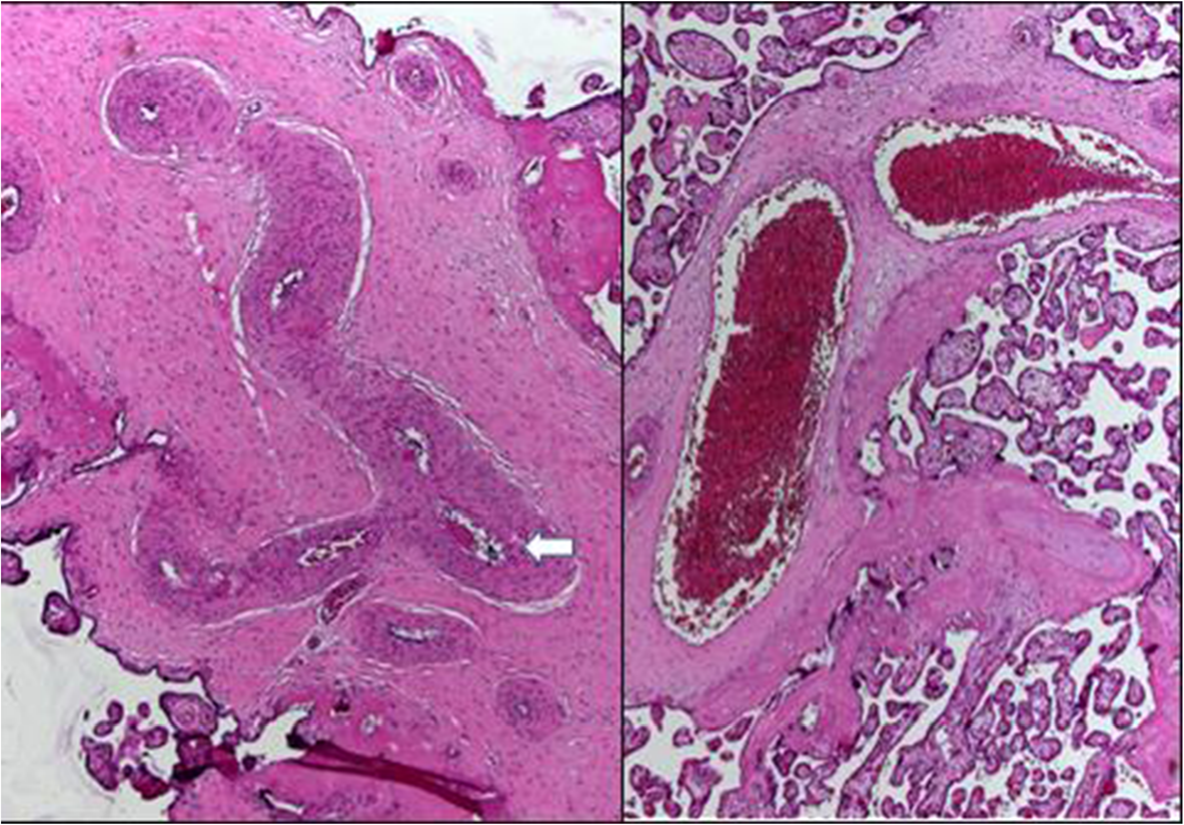
Stem villous vessels with thrombus (*arrow*) and stenosis (*pictured left*). Stem villous vessels normally pervious, for comparison (*pictured right*)

The obstetrician reported difficulties in clamping and cutting the umbilical cord, which appeared edematous and showed signs of clotting and hematoma (Fig. 1). On macroscopic examination, the placenta showed signs of FTV. On histologic examination, thrombosis of several fetal vessels of the chorionic plate and avascular terminal villi, including a macroscopic visible thrombosis of the umbilical artery in an edematous umbilical cord, were demonstrated (Figs. 2 and 3)

**Fig. 4:**
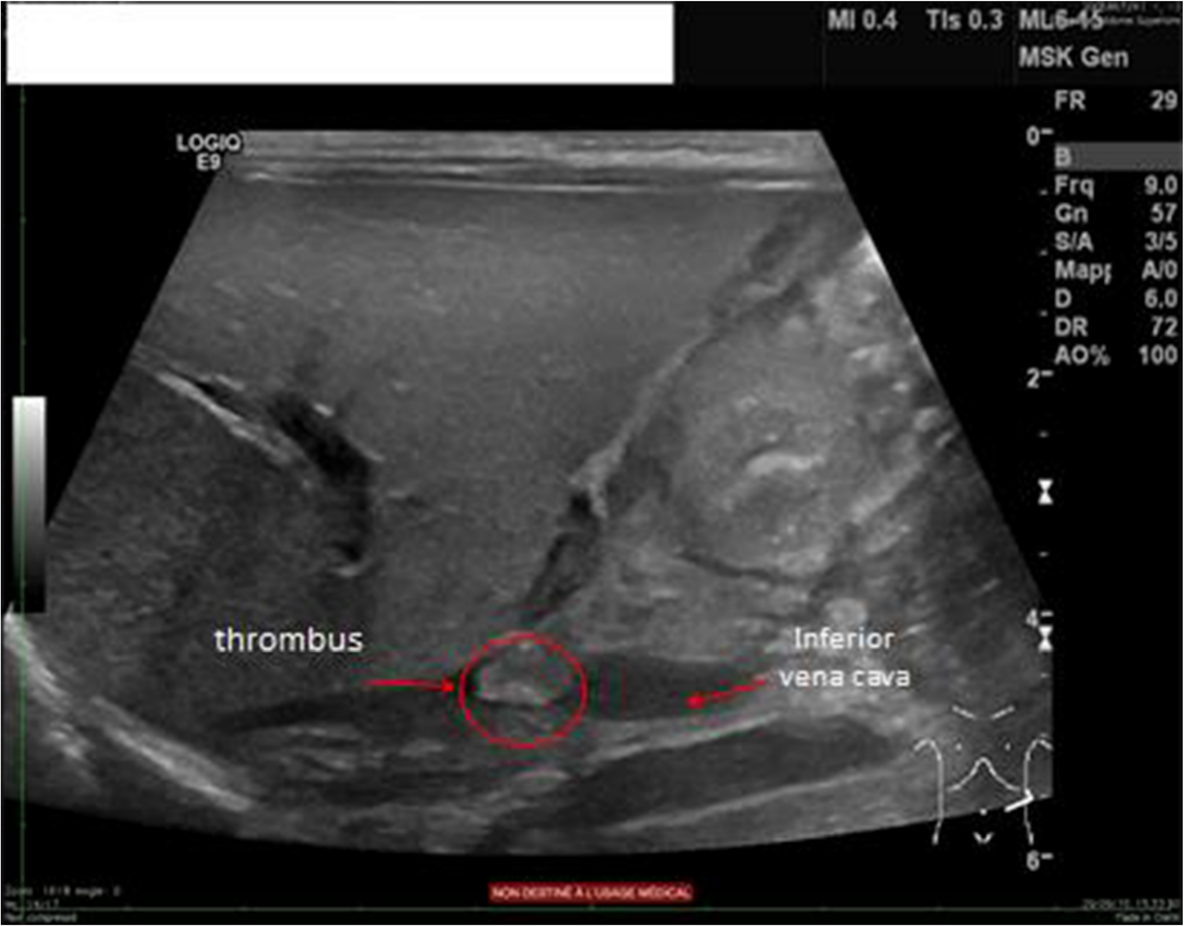
Hyperechoic oval structure (8.5 × 4 mm) at the level of the inferior vena cava near to the opening of the renal veins suggestive of an intraluminal caval thrombus

Despite his normal clinical appearance, an abdominal ultrasound was performed to rule out any conditions potentially related to placental FTV. The ultrasound revealed a hyperechoic oval structure (8.5 × 4 mm) inside his IVC near the conjunction of his renal veins, suggestive of an intraluminal caval thrombus (Fig. 4). Of note, a cranial ultrasonography revealed no abnormalities.

He was admitted to our neonatal division for clinical monitoring and observation. In accordance with the pediatric hematologist, anticoagulation with low-molecular-weight heparin (enoxaparin) at a dose of 1.5 mg/kg twice a day was started, targeting an anti-Xa between 0.5 and 1.0 U/ml [4].

**Fig. 5:**
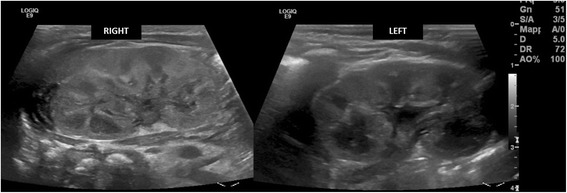
Right kidney parenchyma appears more finely and diffusely hyperechoic than the left

Two days later, a second ultrasonography confirmed the presence of a thrombus of 7.5 × 4 mm, extending into his IVC from his right renal vein. His ipsilateral kidney was slightly more hyperechoic with respect to the left, suggesting parenchymal edema (Fig. 5). Magnetic resonance imaging confirmed the diagnosis. His creatinine reached a maximum value of 127 μmol/L on the same day; this value is high for neonatal age [5] suggesting initial renal vein involvement. His blood pressure was always normal.

**Fig. 6:**
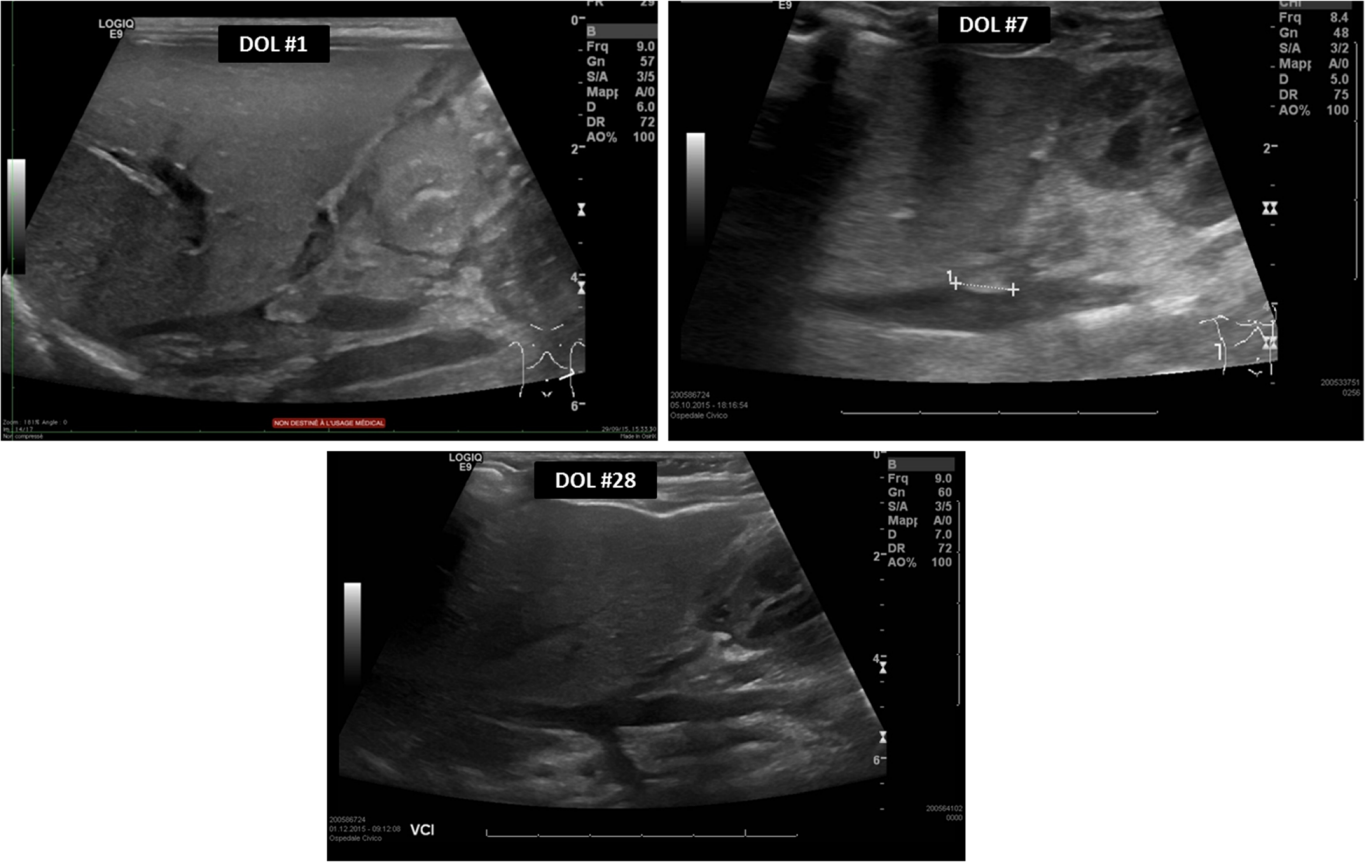
*Top left*: Thrombus at day of life 1. *Top right*: Initial reduction in the size of the thrombus on day of life 7. *Bottom photo*: Complete regression of the thrombus at day of life 28

A third ultrasonography performed at day of life 7 showed a reduction in the size of the thrombus and normalization of his kidney structure. Accordingly, his creatinine decreased to normal values. He was discharged at day of life 8, in good clinical condition and with enoxaparin to be continued for 3 months. Another ultrasound examination at day of life 28 showed an almost complete resolution of the thrombus (Fig. 6).

Thrombophilia screening in his mother showed a prothrombin gene G20210A heterozygous mutation. Thrombophilia screening in the child was performed at 3 months of age with normal results. He was in excellent clinical condition at 1-year follow-up, and is thriving normally.

## Discussion

TEs in infancy, both arterial and venous, are rare but linked with a high risk of morbidity and mortality, which is why several countries are collecting data on these patients in national registries. Neonates have a higher incidence of thrombosis as compared to older children. It has been suggested that neonates are particularly susceptible to such thrombotic complications, as they have decreased levels of anticoagulants and lower levels of fibrinolytic components. The physiologic decrease in activity at different levels of the hemostatic system usually leads to a balance of the prothrombotic and antithrombotic components in healthy neonates.

In the literature, the reported incidence of symptomatic venous TEs ranges from 0.07 to 0.14/10,000 children and is 24/10,000 patients in neonatal intensive care units [6–8]. Several studies based on national and international registries have evaluated the role of risk factors for thrombosis both in children and neonates [6–8]. While the vast majority of venous TEs in neonates and children are associated with the presence of central venous lines, renal vein thrombosis represents the most common non-catheter-related cause of venous TEs occurring during the neonatal period [1]. The etiology of renal vein thrombosis is not precisely known. Reported associated risk factors include perinatal asphyxia, maternal diabetes, and infections [1]. To the best of our knowledge, renal vein thrombosis associated with FTV has not been reported so far.

FTV is a placental abnormality characterized by clusters of not adequately vascularized villi that is often related to upstream thrombosis in placental fetal vessels [9]. On microscopic examination, FTV is diagnosed by the presence of one or more thrombosed fetal vessels. In 2004, the Fetal Vascular Obstruction Nosology Committee formally defined FTV as the presence of 15 or more avascular villi or villous stromal-vascular karyorrhexis in two or more foci per slide, with or without an identifiable fetal vessel lesion and in the absence of “villitis of unknown etiology with stem villitis and avascular villi” [10]. On the basis of this definition, the incidence of FTV in the several studies varies from 1 to 6.4% [11]. The etiology and clinical features of FTV are mostly unknown. Some conditions such as hypercoagulable state, endothelial damage, blood flow stasis, maternal diabetes, and thrombophilia have been associated with FTV [12]. Obstructive cord abnormalities such as excessively long or hypercoiled cord, entanglement, true knots, marginal/membranous insertion, decreased Wharton’s jelly, cord diameter < 8 mm are closely associated with FTV and consequently to neonatal thrombosis [9, 13].

Despite there being no consensus on the causes of FTV, the adverse outcomes of this vasculopathy have been described, raising an evolving concern. Intrauterine growth retardation, fetal demise, intestinal atresia, and thromboembolism into the fetal circulation probably affecting liver, heart, and central nervous system are included [14].

## Conclusions

Our case report stresses two important considerations. First, thrombosis of the renal vein and IVC in our healthy asymptomatic term neonate is clearly associated with FTV. Thus, FTV should be included among the reported causes of renal vein thrombosis in neonates. Furthermore, the incidental nature of the diagnosis of thrombosis in this neonate suggests that unrecognized FTV possibly leads to unrecognized neonatal thrombosis, which in turn may compromise growth and function of the involved organs. Our case underlines the importance of macroscopic examination of the umbilical cord and placenta at delivery. Suspected FTV should lead to microscopic examination of the placenta and ultrasound examination of the neonates to exclude thrombosis of major vessels.

## Abbreviations

FTV: Fetal thrombotic vasculopathy
IVC: Inferior vena cava
TEs: Thromboembolic events
